# SARS-CoV-2 vaccination of convalescents boosts neutralization capacity against Omicron subvariants BA.1, BA.2 and BA.5 and can be predicted by anti-S antibody concentrations in serological assays

**DOI:** 10.3389/fimmu.2023.1170759

**Published:** 2023-04-25

**Authors:** Alina Seidel, Simone Hoffmann, Bernd Jahrsdörfer, Sixten Körper, Carolin Ludwig, Christiane Vieweg, Dan Albers, Pascal von Maltitz, Rebecca Müller, Ramin Lotfi, Patrick Wuchter, Harald Klüter, Frank Kirchhoff, Michael Schmidt, Jan Münch, Hubert Schrezenmeier

**Affiliations:** ^1^ Institute of Molecular Virology, Ulm University Medical Center, Ulm, Germany; ^2^ Institute for Clinical Transfusion Medicine and Immunogenetics Ulm, German Red Cross Blood Transfusion Service Baden- Württemberg-Hessen and University Hospital Ulm and Institute of Transfusion Medicine, University of Ulm, Ulm, Germany; ^3^ Institute of Transfusion Medicine and Immunology, Medical Faculty Mannheim, Heidelberg University; German Red Cross Blood Service Baden-Württemberg– Hessen, Mannheim, Germany; ^4^ Institute of Transfusion Medicine and Immunohematology, German Red Cross Blood Transfusion Service Baden-Württemberg – Hessen, Frankfurt, Germany

**Keywords:** SARS-CoV-2, vaccination, convalescent plasma, neutralization, omicron

## Abstract

**Background:**

Recent data on immune evasion of new SARS-CoV-2 variants raise concerns about the efficacy of antibody-based COVID-19 therapies. Therefore, in this study the *in-vitro* neutralization capacity against SARS-CoV-2 variant B.1 and the Omicron subvariants BA.1, BA.2 and BA.5 of sera from convalescent individuals with and without boost by vaccination was assessed.

**Methods and findings:**

The study included 313 serum samples from 155 individuals with a history of SARS-CoV-2 infection, divided into subgroups without (n=25) and with SARS-CoV-2 vaccination (n=130). We measured anti-SARS-CoV-2 antibody concentrations by serological assays (anti-SARS-CoV-2-QuantiVac-ELISA (IgG) and Elecsys Anti-SARS-CoV-2 S) and neutralizing titers against B.1, BA.1, BA.2 and BA.5 in a pseudovirus neutralization assay. Sera of the majority of unvaccinated convalescents did not effectively neutralize Omicron sublineages BA.1, BA.2 and BA.5 (51.7%, 24.1% and 51.7%, resp.). In contrast, 99.3% of the sera of superimmunized individuals (vaccinated convalescents) neutralized the Omicron subvariants BA.1 and BA.5 and 99.6% neutralized BA.2. Neutralizing titers against B.1, BA.1, BA.2 and BA.5 were significantly higher in vaccinated compared to unvaccinated convalescents (p<0.0001) with 52.7-, 210.7-, 141.3- and 105.4-fold higher geometric mean of 50% neutralizing titers (NT50) in vaccinated compared to unvaccinated convalescents. 91.4% of the superimmunized individuals showed neutralization of BA.1, 97.2% of BA.2 and 91.5% of BA.5 with a titer ≥ 640. The increase in neutralizing titers was already achieved by one vaccination dose. Neutralizing titers were highest in the first 3 months after the last immunization event. Concentrations of anti-S antibodies in the anti-SARS-CoV-2-QuantiVac-ELISA (IgG) and Elecsys Anti-SARS-CoV-2 S assays predicted neutralization capacity against B.1 and Omicron subvariants BA.1, BA.2 and BA.5.

**Conclusions:**

These findings confirm substantial immune evasion of the Omicron sublineages, which can be overcome by vaccination of convalescents. This informs strategies for choosing of plasma donors in COVID-19 convalescent plasma programs that shall select specifically vaccinated convalescents with very high titers of anti-S antibodies.

## Introduction

The B.1.1.529 variant of SARS-CoV-2 was first reported to the World Health Organization from South Africa on 24 November 2021 (1) and has been classified as a variant of concern (VOC), named Omicron (1). Since then, several Omicron subvariants, e.g. BA.1, BA.2 and BA.5, evolved and have been circulating globally (2). The role of passive immune therapy of COVID-19 by convalescent plasma (CCP) is still under investigation. Data suggest efficacy of CCP in early intervention (3–9), in particular among seronegative patients and immunosuppressed patients (10–12). A significant antibody dose response relationship has been observed in some of the CCP trials (4, 5, 13, 14). Omicron might escape passive immune therapy since it can evade neutralization by sera from vaccinated and convalescent individuals and by monoclonal antibodies *in-vitro* (15–21), and the risk of reinfection with Omicron is higher compared to other VOC (15). In this study, we assessed the neutralization capacity against B.1, BA.1, BA.2, and BA.5 of sera from convalescents, some but not all of which were vaccinated. The question was whether superimmunized individuals, i.e. vaccinated convalescents, had cross-neutralization capacity against Omicron sufficient to be considered as plasma donors for passive immune therapy.

## Methods

313 serum samples from 155 individuals with previous SARS-CoV-2 infection (with or without SARS-CoV-2 vaccination) were analyzed by two commercially available assays according to the instructions of the manufacturer (anti-SARS-CoV-2-QuantiVac-ELISA (IgG), Euroimmun, Lübeck, Germany and Elecsys Anti-SARS-CoV-2 S, Roche, Mannheim, Germany). For individuals who have been measured several times, the sera were obtained from independent plasma donations performed at different dates. Samples were collected after written informed consent was obtained from convalescent plasma donors (22) and vaccinated individuals. The studies were approved by the Ethical Committee of University of Ulm and Ethical Committee II, Heidelberg University (392/20, 488/20, 56/21 and 41/22).

### Preparation of pseudotyped particles

Production of rhabdoviral pseudotypes has been previously described (23). In brief, 293T cells (ATCC no. CRL-3216) were transfected with expression plasmids encoding SARS-CoV-2 spike variants B.1 (24), BA.1 (25), BA.2 (26), or BA.5 (27)(kindly provided by Stefan Pöhlmann, Infection Biology Unit, German Primate Center, Göttingen, Germany) by Transit LT-1 (Mirus). One day after transfection, cells were inoculated with a replication-deficient vesicular stomatitis virus (VSV) vector in which the genetic information for its native glycoprotein (VSV-G) is replaced by genes encoding enhanced green fluorescent protein and firefly luciferase (FLuc) (kindly provided by Gert Zimmer, Institute of Virology and Immunology, Mittelhäusern, Switzerland), and incubated for 2 h at 37°C. Then the inoculum was removed, cells were washed with phosphate-buffered saline (PBS) and fresh medium containing anti-VSV-G antibody (I1-hybridoma cells; ATCC no. CRL-2700) was added to block remaining VSV-G carrying particles. After 16-18 h, supernatants were collected and centrifuged (2.000 x g, 10 min, room temperature) to clear cellular debris. Samples were then aliquoted and stored at -80°C.

### SARS-CoV-2 spike pseudovirus neutralization assay

Pseudovirus neutralization experiments were performed as previously described (23). In brief, Vero E6 cells were seeded in 96-well plates one day prior (6000 cells/well, 2.5% FCS) infection. Sera were heat-inactivated (56°C, 30 min) and serially titrated (4-fold titration series with 7 steps + buffer only control) in PBS, undiluted pseudovirus stocks added (1:1, v/v) and the mixtures incubated for 30 min at 37°C before being added to cells in duplicates (final on-cell dilution of sera: 20, 80, 320, 1280, 5120, 20480, 81920-fold). After an incubation period of 16-18 h, transduction efficiency was analyzed. For this, the supernatant was removed, and cells were lysed by incubation with Cell Culture Lysis Reagent (Promega) at room temperature. Lysates were then transferred into white 96-well plates and luciferase activity was measured using a commercially available substrate (Luciferase Assay System, Promega) and a plate luminometer (Orion II Microplate Luminometer, Berthold). For analysis of raw values [relative luminescence units per s (RLU/s)], background signal of untreated cells was subtracted and values normalized to cells inoculated with pseudovirus preincubated with PBS only. Results are given as serum dilution on cell resulting in 50% pseudovirus neutralization (NT50), calculated by nonlinear regression ([Inhibitor] vs. normalized response – Variable slope) in GraphPad Prism Version 9.1.1. According to the serum dilution factors tested, the upper and lower cutoff value of the assay was 81920 and 20, respectively. For quantitative analyses, NT50 values <20 were set to a value of 10.

### Statistical analyses

The p-values for the pairwise comparisons were calculated by Kruskal-Wallis Test. Statistical significance between more than two groups was evaluated using Kruskal-Wallis test followed by Dunn’s Test as correction for multiple comparisons, as described in the figure legends. Correlations were assessed using Spearman correlation analysis. A p value of less than 0.05 was considered statistically significant. Statistical analyses were performed using GraphPad Prism Version 9.0.2, GraphPad Software, San Diego, California USA, www.graphpad.comand NCSS 2021 Statistical Software (2021). NCSS, LLC. Kaysville, Utah, USA, ncss.com/software/ncss.

## Results

We studied 313 serum samples from a cohort of 155 individuals with a history of SARS-CoV-2 infection (**Table 1**). The cohort has been subdivided in a group without vaccination (n=25) and a group with vaccination (n=130).

**Table 1:** Characteristics of the study cohort of convalescent individuals.

	Prior history of infection no vaccination (n=25)	Prior history of infection + vaccination (n=130)
Median age, years (IQR)	47 (31.5-57)	41.5 (28-53)
Gender, no		
Female/male	11/14	71/60
Median interval since infection, days (IQR)	117.0 (85-131,5)	105 (58-325)
Variant (time of infection as proxy), n (%)		
B.1	25 (100)	37 (28.5)
Alpha	–	6 (4.6)
Delta	–	40 (30.8)
BA.1	–	27 (20.8)
BA.2	–	18 (13.8)
unknown	–	2 (1.5)
No. of vaccination doses, n (%) 0	25 (100%)	–
1	–	19 (14.6)
2	–	41 (31.5)
3	–	70 (53.8)
Vaccination regimen, n (%)		
Heterologous	–	40 (30.8)
Homologous	–	90 (69.2)
Vaccines homologous regimen, n (%)	n.a.	
BNT162b		74 (82.2)
mRNA-1273		10 (11.1)
ChAdOx1		5 (5.6)
COVID-19 vaccine Janssen		1 (1.1)
Vaccines heterologous regimen, n (%)	n.a.	
ChAdOx1/BNT162b		1 (2.5)
ChAdOx1/BNT162b/BNT162b		5 (12.5)
ChAdOx1/BNT162b/mRNA-1273		1 (2.5)
ChAdOx1/mRNA-1273/mRNA-1273		2 (5)
BNT162b/BNT162b/mRNA-1273		18 (45)
mRNA-1273/BNT162b		1 (2.5)
mRNA-1273/mRNA-1273/BNT162b		6 (15)
ChAdOx1/ChAdOx1/BNT162b		1 (2.5)
ChAdOx1/BNT162b/mRNA-1273		1 (2.5)
COVID-19 vaccine		1 (2.5)
Janssen/BNT162b/mRNA-1273		2 (5)
COVID-19 vaccine Janssen/mRNA-1273		1 (2.5)
COVID-19 vaccine Janssen/BNT162b/BNT162b		
Median interval since last vaccination	n.a.	126.5 (57 – 188)

n.a., not applicable.

**Figure 1:**
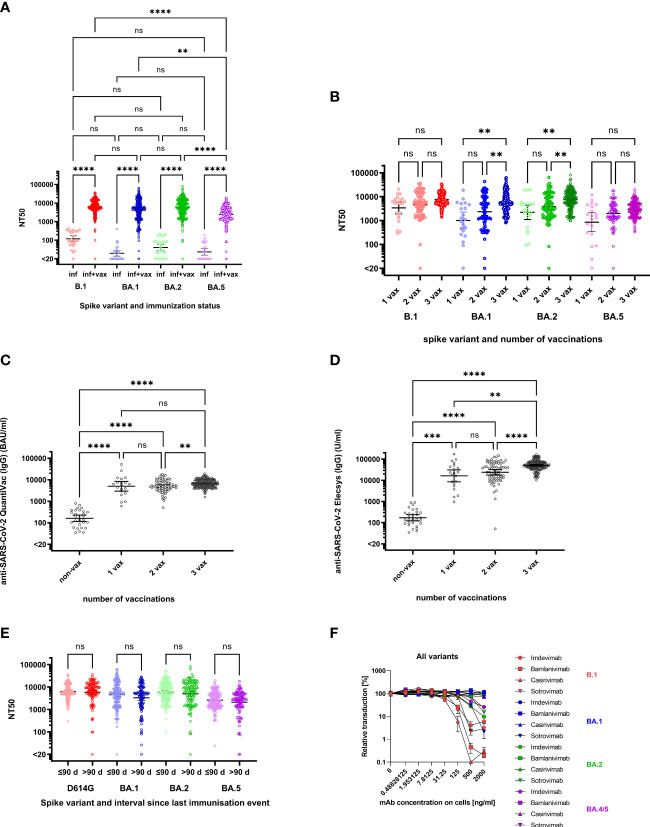
Neutralization of Spike variants by convalescent sera and monoclonal antibodies. **(A)** NT50 against B.1 (red symbols), BA.1 (blue symbols), BA.2 (green symbols) and BA.5 (purple symbols) for individuals with a history of infection (inf, lighter colors) (n=25) and history of infection and vaccination (inf + vax, darker colors) (n=130). The geometric means of NT50 were as follows: against B.1 (inf) 118.4, B.1 (inf+vax) 6247, BA.1 (inf) 19.56, BA.1 (inf+vax) 4122, BA.2 (inf) 40.41, BA.2 (inf+vax) 5709, BA.5 (inf) 23.24 and against BA.5 (inf+vax) 2449. Neutralization of Omicron sublineages BA.1, BA.2 and BA.5 was observed in 51.7%, 24.1% and 51.7% resp., of convalescent individuals without vaccination. In contrast, 99.3% of the sera of superimmunised individuals (vaccinated convalescents) neutralized the Omicron subvariants BA.1 and BA.5 and 99.6% neutralized BA.2. **(B)** NT50 against B.1 (red), BA.1 (blue), BA.2 (green) and BA.5 (purple) for vaccinated, convalescent donors stratified by number of vaccination doses: 1 vaccination dose (1 vax, lighter colors) (n=19), 2 vaccination doses (2 vax, medium light colors) (n=41) and 3 vaccination doses (3 vax, darker colors) (n=71). The geometric means of NT50 were as follows: against B.1 3389 (1 vax), 4610 (2 vax), 7546 (3 vax), against BA.1 1005 (1 vax) and 2347 (2 vax), 6073 (3 vax), against BA.2 2208 (1 vax) and 3859 (2 vax), 7448 (3 vax) and against BA.5 852 (1 vax), 1999 (2 vax), 2.973 (3 vax). **(C)** Anti-SARS-CoV-2-QuantiVac (IgG) titers of convalescent donors stratified by number of vaccinations. The geometric means were as follows: non-vax 161.1 BAU/ml, 1 vax 5000 BAU/ml, 2 vax 5023 BAU/ml and 3 vax 6749 BAU/ml. **(D)** Anti-SARS-CoV-2 Elecsys (IgG) titers of convalescent donors stratified by number of vaccinations. The geometric means were as follows: 171 U/ml for non-vax, 16262 U/ml for 1 vax, 23913 U/ml for 2 vax and 51127 U/ml for 3 vax. **(E)** NT50 against B.1 (red), BA.1 (blue), BA.2 (green) and BA.5 (purple) for vaccinated, convalescent donors stratified by interval between last immunization event and collection of serum sample: ≤ 90 days (lighter colors) (n=113) and >90 days (darker colors) (n=171). The geometric means of NT50 were as follows: against B.1 6226 (≤90 days) and 5841 (>90 days), against BA.1 4545 (≤90 days) and 3302 (>90 days), against BA.2 5928 (≤90 days) and 5051 (>90 days) and against BA.5 2593 (≤90 days) and 2102 (>90 days). **(F)** Inhibition of cell entry of B.1 (red symbols), BA.1 (blue symbols), BA.2 (green symbols) and BA.5 (purple symbols) spike carrying pseudoparticles by monoclonal antibodies. Increasing doses of Bamlanivimab (squares), Casirivimab (up-pointing triangles), Sotrovimab (down-pointing triangles) and Imdevimab (circles) were preincubated with pseudoparticles before addition to cells (doses were titrated in 4-fold dilution from 2000 ng/ml to 0.49 ng/ml (referring to final concentrations on cells)). Infection rates in Figures **(A,B,E,F)** were determined 16 hours post infection by measuring luciferase activity in cellular lysates. Data shown were derived from one experiment performed in duplicates. The p-values for the pairwise comparisons shown in **(A–D)** were calculated by Kruskal-Wallis Test (not significant (ns) p>0.05, ** p<0.01, *** p<0.001, **** p<0.0001). For Figure **(B)** Kruskal-Wallis Test was followed by Dunn’s test for correction for multiple comparisons. The horizontal black lines denote the geometric mean of NT50 and the error bars the 95%-confidence interval of the geometric mean.

Non-vaccinated individuals with a history of infection exhibited B.1 neutralizing titers of 118 (geometric mean neutralizing (GMN) titers, 95% confidence interval (95%-CI) 81-174) (**Figure 1A**). Neutralization of Omicron BA.1, BA.2 and BA.5 was undetectable (i.e. below a titer of 20) in 15/29 (51.7%), 7/29 (24.1%) and 15/29 (51.7%) of convalescent individuals with GMN titers of 19 (14–28), 40 (27–60) and 23 (16–34). However, convalescents who had received at least one vaccination dose exhibited significantly higher neutralizing titers compared to non-vaccinated convalescents even though their NT50 against BA.5 (2449, 2164-2771) was lower than against B.1 (6246, 5607-6959), BA.1 (4122, 3519-4828) and BA.2 (5708, 4988-6534) (**Figure 1A**). Fold-difference in GMN titers of vaccinated versus non-vaccinated convalescents was 52.7-fold for B.1, 211-fold for BA.1, 141-fold for BA.2 and 105-fold for BA.5 (**Figure 1A**). Neutralizing titers against B.1, BA.1 and BA.2 did no longer differ significantly in convalescents after vaccination (**Figure 1A**). Only three of the superimmunized individuals did not neutralize Omicron variants. One individual was completely unable to neutralize all SARS-CoV-2 variants. Another individual, most likely infected with B.1, could not neutralize BA.1 and the neutralization for BA.5 was very close to the cut-off (NT50 of 23.94). In general, the NT50 values of this individual were not very high: NT50 against B.1 and BA.2 were 289 and 95.42. In contrast, the third individual had NT50 values of 470.5 and 1458 against BA.1 and BA.2 but did not neutralize BA.5.

Already one dose of vaccination in convalescent individuals was sufficient to drastically increase their NT50 values (26.6-fold increase for B.1, for Omicron BA.1, BA.2, and BA.5 an increase of 51.4-, 54.6-, and 53.9-fold was measured). A higher number of vaccinations yielded a further increase of NT50 (**Figure 1B**) with significant increase after 3 vaccinations compared to only one vaccination for BA.1 and BA.2. For B.1 and BA.5, neutralizing titers were not significantly different between subjects who received either one, two or three vaccinations, and for BA.1 and BA.2, differences between neutralizing titers were only significant for some of the comparisons: the increase from one vaccination compared to three vaccinations, and the increase from two vaccinations compared to three vaccinations (**Figure 1B**). A similar development was observed when comparing IgG titers with number of vaccinations: One dose of vaccination led to a 31-fold increase in anti-SARS-CoV-2 antibody concentrations (non-vax: 161.1 BAU/ml; 1 vax: 5000 BAU/ml, quantified *via* anti-SARS-CoV-2-QuantiVac-ELISA) but a second dose did not significantly change antibody concentrations (5023 BAU/ml). However, a third vaccination significantly improved anti-SARS-CoV-2 antibody titers (6749 BAU/ml) (**Figure 1C**). A similar trend was also obtained with the Elecsys Anti-SARS-CoV-2 S ELISA (**Figure 1D**). A more detailed comparison in terms of descriptive statistics can be found in **Table 2**.

**Table 2:** Comparison IgG titers measured by anti-SARS-CoV-2 Quantivac and Elecsys with number of vaccinations.

	QuantiVac (IgG) (BAU/ml)				Elecsys (IgG) (U/ml)			
	Non-vax	1 vax	2 vax	3 vax	Non-vax	1 vax	2 vax	3 vax
Min.	35	595.5	510.8	1582	34.40	986.0	51.80	10279
Max.	801.7	52199	17639	18951	902	172400	148810	166430
Range	766.7	51604	17128	17370	867.6	171432	148758	156151
25% percentile	71.73	2501	3522	4934	92	6105	12442	39170
75% percentile	361.8	10210	7935	9664	309	47596	62169	72900
Geo. mean	161.1	5000	5023	6749	171	16262	23913	51127

Neutralizing titers against all variants were higher, although not significant, among those with an interval ≤90 days since the last immunization event compared to intervals >90 days. The geometric means of NT50 against B.1 were 6226 (≤90 days) and 5841 (>90 days), against BA.1 4545 (≤90 days) and 3302 (>90 days), BA.2 5928 (≤90 days) and 5051 (>90 days), and against BA.5 2593 (≤90 days) and 2102 (>90 days) (**Figure 1E**).

As a control, we also investigated the neutralizing capacity of several monoclonal antibodies. While variants BA.1, BA.2, and BA.5 were neutralized by the polyvalent antibodies of convalescent, vaccinated individuals (**Figure 1A**), they were mostly resistant against the monoclonal antibodies Bamlanivimab, Casirivimab and Imdevimab, as previously reported (25, 28, 29). Only Sotrovimab neutralized all tested variants (25) (**Figure 1F**).

**Figure 2:**
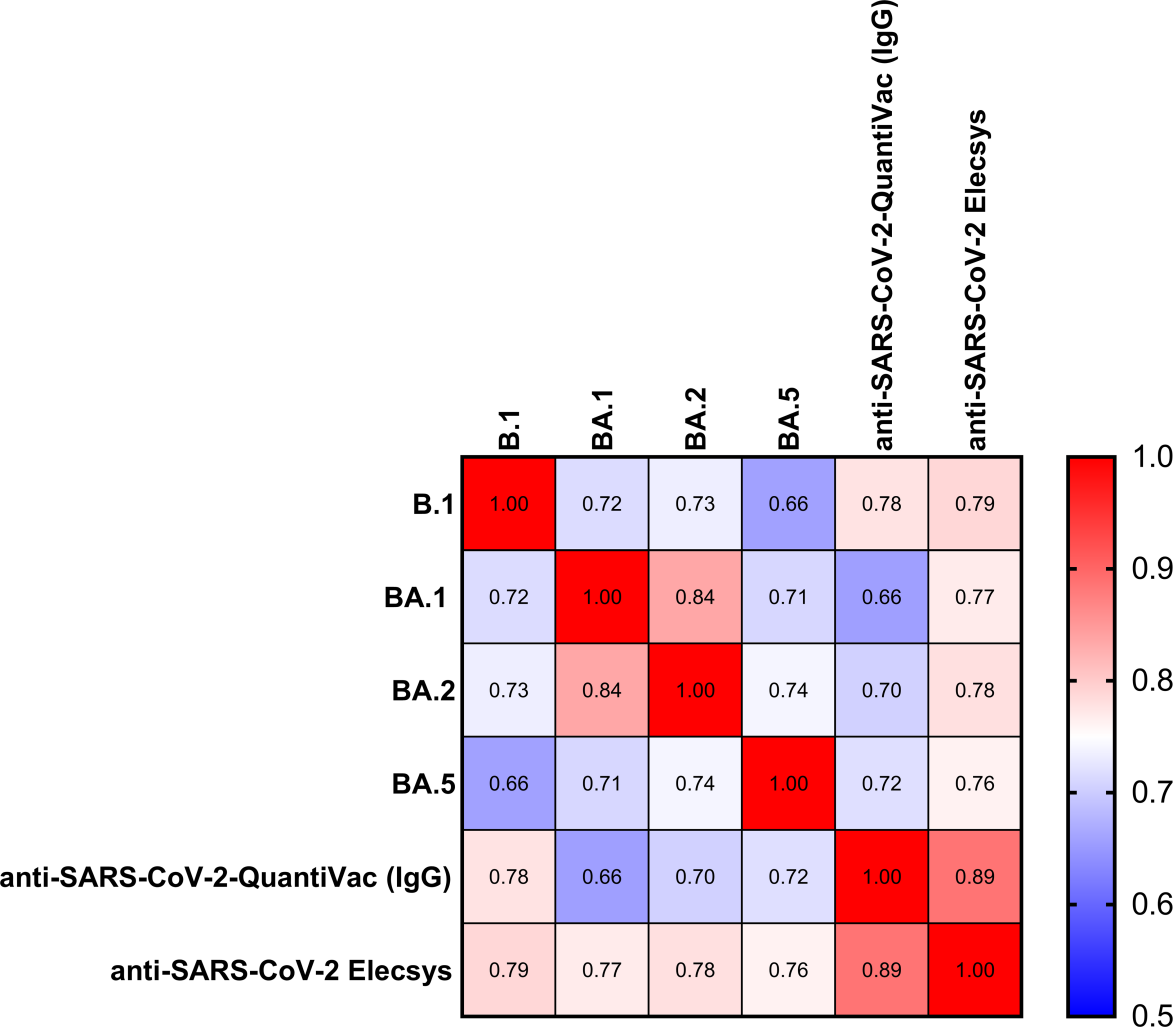
Correlation of anti-S antibody concentrations and neutralization capacity against spike variants. Correlation matrix of NT50 against BA.1, BA.2, BA5 and B.1, and anti-SARS-CoV-2-QuantiVac (IgG) ELISA and Elecsys SARS-CoV-2 based on Spearman Correlation.

**Figure 3:**
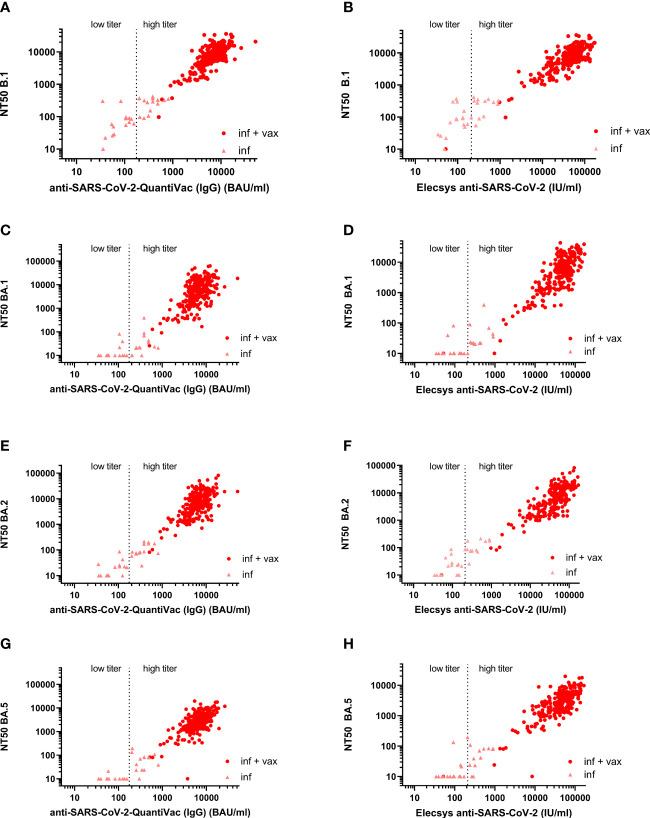
Correlation between anti-S antibody concentrations and NT50 against B.1, BA.1, BA.2, and BA.5. **(A)** Correlation between anti-S antibody concentrations measured by anti-SARS-CoV-2-QuantiVac-ELISA (IgG) and NT50 against B.1 (Spearman correlation 0.78). **(B)** Correlation between anti-S antibody concentrations measured by Elecsys Anti-SARS-CoV-2 S and NT50 against B.1 (Spearman correlation 0.79). **(C)** Correlation between anti-S antibody concentrations measured by anti-SARS-CoV-2 QuantiVac-ELISA (IgG) and NT50 against BA.1 (Spearman correlation 0.66). **(D)** Correlation between anti-S antibody concentrations measured by Elecsys Anti-SARS-CoV-2 S and NT50 against BA.1 (Spearman correlation 0.77). **(E)** Correlation between anti-S antibody concentrations measured by anti-SARS-CoV-2-QuantiVac-ELISA (IgG) and NT50 against BA.2 (Spearman correlation 0.70). **(F)** Correlation between anti-S antibody concentrations measured by Elecsys Anti-SARS-CoV-2 S and NT50 against BA.2 (Spearman correlation 0.78). **(G)** Correlation between anti-S antibody concentrations measured by anti-SARS-CoV-2 QuantiVac-ELISA (IgG) and NT50 against BA.5 (Spearman correlation 0.72). **(H)** Correlation between anti-S antibody concentrations measured by Elecsys Anti-SARS-CoV-2 S and NT50 against BA.5 (Spearman correlation 0.76). Figures **(A–H)** Results of non-vaccinated convalescents (inf) are shown as triangles, and results of vaccinated convalescents (inf + vax) are shown as filled circles. The vertical dashed line at 176 BAU/ml for the anti-SARS-CoV-2 QuantiVac and at 210 U/ml for the Elecsys anti-SARS-CoV-2 represents the threshold above which CCP is considered high-titer CCP according to the FDA’s recommendations for investigational CCP (30).

**Figure 4:**
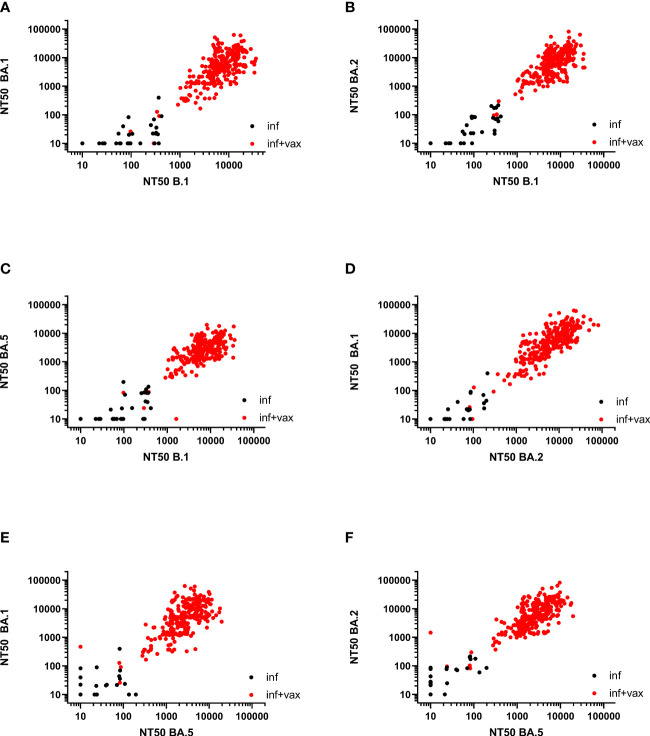
Correlation between NT50 values of SARS-CoV-2 variants. **(A)** Correlation between NT50 against BA.1 and NT50 against B.1 (Spearman correlation 0.72). **(B)** Correlation between NT50 against BA.2 and NT50 against B.1 (Spearman correlation 0.73). **(C)** Correlation between NT50 against BA.5 and NT50 against B.1 (Spearman correlation 0.66). **(D)** Correlation between NT50 against BA.1 and NT50 against BA.2 (Spearman correlation 0.84). **(E)** Correlation between NT50 against BA.1 and NT50 against BA.5 (Spearman correlation 0.71). **(F)** Correlation between NT50 against BA.2 and NT50 against BA.5 (Spearman correlation 0.74).

The Spearman correlation (SC) matrix of NT50 against B.1 and respective Omicron subvariants, the anti-SARS-CoV-2-QuantiVac-ELISA (IgG) and the Elecsys Anti-SARS-CoV-2 S revealed good correlations between all assays, in particular between the two anti-SARS-CoV-2 serological assays (SC 0.89) and between the NT50 against BA.1 and BA.2 (SC 0.84) (**Figure 2**). SCs between anti-SARS-CoV-2-QuantiVac-ELISA (IgG) and NT50 against Omicron BA.1, BA.2 and BA.5 were 0.66, 0.70 and 0.72 (**Figures 3A, C, E, G**). The SC between Elecsys Anti-SARS-CoV-2 S and NT50 against BA.1, BA.2 and BA.5 were 0.77, 0.78 and 0.76 (**Figures 3B, D, F, H**). The SCs between the NT50 values of the respective subvariants show good correlations: the SCs between NT50 against B.1 and NT50 against Omicron BA.1, BA.2 and BA.5 were 0.72, 0.73 and 0.66, respectively (**Figures 4A-C**). The SCs between NT50 against BA.1 and NT50 against BA.2 and BA.5 were 0.84 and 0.71 (**Figures 4D, E**). SCs between NT50 against BA.2 and NT50 against BA.5 was 0.74 (**Figures 4F**). This indicates that superimmunized individuals can cover novel variants.

**Figure 5:**
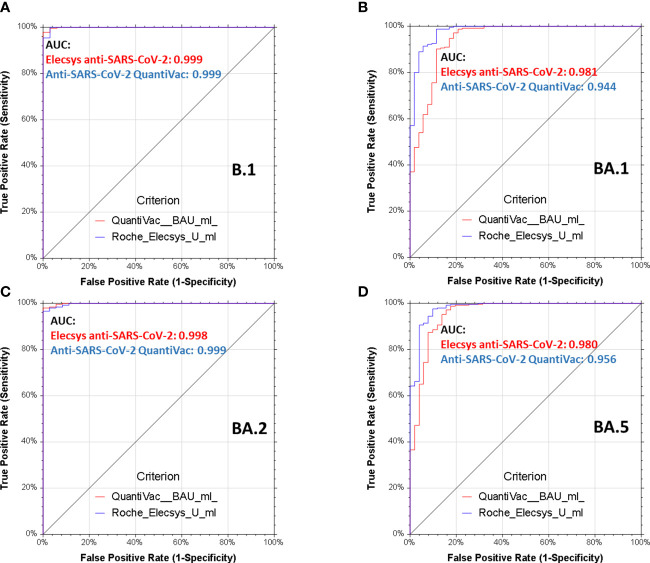
Receiver operating characteristics analyses of serological assays and neutralization of spike variants. Receiver operating characteristics (ROC) analyses of Elecsys Anti-SARS-CoV-2 S (red lines) and anti-SARS-CoV-2-QuantiVac-ELISA (IgG) (blue lines) prediction of neutralization of B.1 **(A)**, BA.1 **(B)**, BA.2 **(C)** and BA.5 **(D)**. A positive neutralizing titer was arbitrarily defined as ≥640. Area under the curve (AUC) is reported in the graphs, p<0.0001 for all serological assays and all spike variants.

Plasma units for immune therapy shall have very high neutralizing titers and based on the outcomes of the CAPSID trial, we adopted NT50≥640 (13, 22, 31). Receiver operating characteristics (ROC) for BA.1, BA.2 and BA.5 demonstrate that both anti-SARS-CoV-2-QuantiVac-ELISA (IgG) and Elecsys anti-SARS-CoV-2 S excellently predict these neutralizing titers with areas under the curve between 0.94, 0.99 and 0.95 for anti-SARS-CoV-2-QuantiVac-ELISA (IgG) and between 0.98 and 0.99 and 0.98 for Elecsys Anti-SARS-CoV-2 S (**Figure 5**).

## Discussion

Significant immune evasion by Omicron has raised concerns that antibody-based therapies may no longer be effective against Omicron variants (25, 28, 29). Here, we focused on convalescent individuals and the implication of evasion from antibody-mediated neutralization for future CCP programs. There is growing evidence that CCP can be an important component in the therapeutic armamentarium for COVID-19 if it is given early and at very high dose (i.e. with high antibody content) to vulnerable patients who are at risk of progression to severe COVID-19 (3, 6, 9, 32). The precise threshold values necessary for a sample to qualify as high-titer convalescent plasma for use in CCP therapy cannot be determined based on the existing evidence, as no dose-response studies have been conducted to establish these criteria. The neutralizing titers of CCPs used in clinical trials has either not been reported or titers have been measured with different (in-house) assays, the results of which are difficult to compare between different trial centers (33). Antibody-negative or immunocompromised recipients are more likely to benefit from CCP. Among hospitalized patients who lacked SARS-CoV-2 antibodies at baseline, CCP decreased the need for mechanical ventilation or mortality compared with standard of care or placebo (8, 34–38). There is evidence for efficacy of CCP in immunocompromised patients both from cohort studies and subgroup analyses of randomized clinical trials (10, 12, 39–42). Further, several CCP studies have demonstrated a dose effect (3, 4, 13, 31, 43, 44). Therefore, in the recent clinical practice guidelines from the Association for the Advancement of Blood and Biotherapies (AABB), CCP was recommended for outpatients with COVID-19 who are at high risk for disease progression, for hospitalized patients with COVID-19 and pre-existing immunosuppression and for hospitalized patients who do not have SARS-CoV-2 antibodies detected at baseline (8). However, this evidence is based on clinical trials which were conducted before the emergence of Omicron. This raised concerns that new variants might escape immunotherapy with CCP. Our data confirm *in-vitro* resistance of Omicron to several monoclonal antibodies used in clinical practice (16–19) questioning their efficacy in Omicron infected patients (28). Also, Omicron BA.1, BA.2 and BA.5 are no longer well neutralized *in-vitro* by sera of convalescents from the first and second surge of the SARS-CoV-2 pandemic. However, in convalescents just one dose of SARS-CoV-2 vaccination restores *in-vitro* neutralization capacity against Omicron. In contrast to other recent reports on significantly lower neutralization capacity in vaccinated convalescent donors (17–19) against Omicron compared to wild type, we observed a similar neutralization capacity against B.1 and Omicron BA.1 and BA.2. This might be due to our high sample size, the neutralization assay, the large proportion of donors with high anti-S antibody concentration in our cohort and the vaccination scheme. Several studies have demonstrated a strong correlation of VSV-based SARS-CoV-2 Spike pseudovirus neutralization assays and the live virus neutralization (45, 46). We did observe lower neutralization capacity against Omicron BA.5 in superimmunized individuals compared to B.1. However, a subgroup of superimmunized individuals still had strong neutralizing activity also against Omicron BA.5. The geometric mean of NT50 of the upper quartile was 7059. Thus, in contrast to monoclonal antibodies, which mostly lost their activity against new SARS-CoV-2 variants (16–19, 47), CCP with very good *in vitro* neutralization capacity can still be obtained. Our findings suggest that even without adaption of currently available vaccines, the broader immune repertoire in superimmunized individuals can cover novel variants (48), particularly in the first three months after the last immunization event when the highest neutralizing titers are achieved.

The neutralization titers in superimmunized individuals are highly variable. For BA.2 the geometric means of the lower and upper quartile were 282 and 19728, resp., and for BA.5 the geometric means of the lower and upper quartile were 148 and 7059, resp., i.e. about a 50-70-fold difference. Thus, for CCP programs it is key to perform a systematic screening of convalescent, vaccinated donors. Here we demonstrate a good correlation between commercially available high-throughput serological assays (Anti-SARS-CoV-2-QuantiVac-ELISA (IgG); Elecsys Anti-SARS-CoV-2 S) and neutralization titers. Thus, these high-throughput serological assays can be used to identify plasma donors with very high SARS-CoV-2 antibody concentrations, who also have very high *in-vitro* neutralizing titers against B.1 and Omicron BA.1, BA.2 and BA.5. Therefore, for future convalescent plasma programs, priority should be given to superimmunized donors with previous infection plus at least one dose of a SARS-CoV-2 vaccination with very high SARS-CoV-2 antibody concentrations as measured by serological assays.

By selection of recently immunized donors with very high concentrations of anti-SARS-CoV-2 antibody concentrations it is possible to generate CCP for passive immunotherapy which is adaptive to viral evolution. A concept of early, very high titer CCP from highly selected superimmunized donors in an era dominated by new variants must be investigated in clinical trials (e.g. the ongoing COVIC-19 trial, EudraCT 2021-006621-22; NCT05271929).

## Data availability statement

The original contributions presented in the study are included in the article/supplementary materials. Further inquiries can be directed to the corresponding author.

## Ethics statement

The studies involving human participants were reviewed and approved by Ethical Committee of University of Ulm, Ulm, Germany. The patients/participants provided their written informed consent to participate in this study.

## Author contributions

HS, SK, and JM designed and supervised research, AS, PvM, DA, FK, and JM performed, analyzed and supervised the neutralization tests; CL, CV, and BJ performed, analyzed and supervised the QuantiVac measurements; MS performed and analyzed the Elecsys measurements; SK, PW, RM, RL, HK, and HS provided samples and clinical data; SH, AS, JM, and HS coordinated the project and analyzed and interpreted the data and wrote the manuscript. All authors contributed to the article and approved the submitted version.

## References

[1] WHO classification of omicron (B.1.1.529): SARS-CoV-2 variant of concern (2021). Available at: https://www.who.int/news/item/26-11-2021-classification-of-omicron-(b11529)-sars-cov-2-variant-of-concern.

[2] Tegally H Moir M Everatt J Giovanetti M Scheepers C Wilkinson E. Emergence of SARS-CoV-2 omicron lineages BA.4 and BA.5 in south Africa. Nat Med (2022) 28(9):1785–90. doi: 10.1038/s41591-022-01911-2 PMC9499863 35760080

[3] Libster R Perez MG Wappner D Coviello S Bianchi A Braem V. Early high-titer plasma therapy to prevent severe covid-19 in older adults. N Engl J Med (2021) 384(7):610–8. doi: 10.1056/NEJMoa2033700 PMC7793608 33406353

[4] Joyner MJ Carter RE Senefeld JW Klassen SA Mills JR Johnson PW. Convalescent plasma antibody levels and the risk of death from covid-19. N Engl J Med (2021) 384(11):1015–27. doi: 10.1056/NEJMoa2031893 PMC7821984 33523609

[5] Salazar E Christensen PA Graviss EA Nguyen DT Castillo B Chen J. Significantly decreased mortality in a Large cohort of coronavirus disease 2019 (COVID-19. patients transfused early with convalescent plasma containing high-titer anti-severe acute respiratory syndrome coronavirus 2 (SARS-CoV-2. spike protein IgG. Am J Pathol (2021) 191(1):90–107. doi: 10.1016/j.ajpath.2020.10.008 33157066 PMC7609241

[6] Sullivan DJ Gebo KA Shoham S Bloch EM Lau B Shenoy AG. Early outpatient treatment for covid-19 with convalescent plasma. N Engl J Med (2022) 386(18):1700–11. doi: 10.1056/NEJMoa2119657 PMC9006786 35353960

[7] de Candia P Prattichizzo F Garavelli S La GR De RA Pontarelli A. Effect of time and titer in convalescent plasma therapy for COVID-19. iScience (2021) 24(8):102898. doi: 10.1016/j.isci.2021.102898 34316549 PMC8297982

[8] Estcourt LJ Cohn CS Pagano MB Iannizzi C Kreuzberger N Skoetz N. Clinical practice guidelines from the association for the advancement of blood and biotherapies (AABB): COVID-19 convalescent plasma. Ann Intern Med (2022) 175(9):1310–21. doi: 10.7326/M22-1079 PMC9450870 35969859

[9] Levine AC Fukuta Y Huaman MA Ou J Meisenberg BR Patel B. COVID-19 convalescent plasma outpatient therapy to prevent outpatient hospitalization: a meta-analysis of individual participant data from five randomized trials. medRxiv (2022). doi: 10.1101/2022.12.16.22283585 PMC10273382 36809473

[10] Thompson MA Henderson JP Shah PK Rubinstein SM Joyner MJ Choueiri TK. Association of convalescent plasma therapy with survival in patients with hematologic cancers and COVID-19. JAMA Oncol (2021) 7(8):1167–75. doi: 10.1001/jamaoncol.2021.1799 PMC8377563 34137799

[11] Senefeld JW Klassen SA Ford SK Senese KA Wiggins CC Bostrom BC. Use of convalescent plasma in COVID-19 patients with immunosuppression. Transfusion (2021) 61(8):2503–11. doi: 10.1111/trf.16525 PMC8242637 34036587

[12] Hueso T Godron AS Lanoy E Pacanowski J Levi LI Gras E. Convalescent plasma improves overall survival in patients with b-cell lymphoid malignancy and COVID-19: a longitudinal cohort and propensity score analysis. Leukemia (2022) 36(4):1025–34. doi: 10.1038/s41375-022-01511-6 PMC8805670 35105946

[13] Körper S Weiss M Zickler D Wiesmann T Zacharowski K Corman VM. Results of the CAPSID randomized trial for high-dose convalescent plasma in patients with severe COVID-19. J Clin Invest (2021) 131(20):e152264. doi: 10.1172/JCI152264 34464358 PMC8516466

[14] O'Donnell MR Grinsztejn B Cummings MJ Justman JE Lamb MR Eckhardt CM. A randomized double-blind controlled trial of convalescent plasma in adults with severe COVID-19. J Clin Invest (2021) 131(13):e150646. doi: 10.1172/JCI150646 33974559 PMC8245169

[15] Rossler A Riepler L Bante D von LD Kimpel J. SARS-CoV-2 omicron variant neutralization in serum from vaccinated and convalescent persons. N Engl J Med (2022) 386(7):698–700. doi: 10.1056/NEJMc2119236 35021005 PMC8781314

[16] Wilhelm A Widera M Grikscheit K Toptan T Schenk B Pallas C. Limited neutralisation of the SARS-CoV-2 omicron subvariants BA.1 and BA.2 by convalescent and vaccine serum and monoclonal antibodies. EBioMedicine (2022) 82:104158. doi: 10.1016/j.ebiom.2022.104158 35834885 PMC9271884

[17] Liu L Iketani S Guo Y Chan JF-W Wang M Liu L. Striking antibody evasion manifested by the omicron variant of SARS-CoV-2. Nature (2022) 602(7898):676–81. doi: 10.1038/d41586-021-03826-3 35016198

[18] Planas D Saunders N Maes P Guivel-Benhassine F Planchais C Buchrieser J. Considerable escape of SARS-CoV-2 omicron to antibody neutralization. Nature (2022) 602, 671–5. doi: 10.1038/d41586-021-03827-2 35016199

[19] Cameroni E Bowen JE Rosen LE Saliba C Zepeda SK Culap K. Broadly neutralizing antibodies overcome SARS-CoV-2 omicron antigenic shift. Nature (2022) 602(7898):664–70. doi: 10.1038/d41586-021-03826-3 PMC9531318 35016195

[20] Wang Q Iketani S Li Z Liu L Guo Y Huang Y. Alarming antibody evasion properties of rising SARS-CoV-2 BQ and XBB subvariants. Cell (2023) 186(2):279–86. doi: 10.1016/j.cell.2022.12.018 PMC9747694 36580913

[21] Wang Q Guo Y Iketani S Nair MS Li Z Mohri H. Antibody evasion by SARS-CoV-2 omicron subvariants BA.2.12.1, BA.4 and BA.5. Nature (2022) 608(7923):603–8. doi: 10.1038/s41586-022-05053-w PMC9385487 35790190

[22] Körper S Jahrsdörfer B Corman VM Pilch J Wuchter P Blasczyk R. Donors for SARS-CoV-2 convalescent plasma for a controlled clinical trial: donor characteristics, content and time course of SARS-CoV-2 neutralizing antibodies. Transfusion Med Hemother (2021) 48(3):137–47. doi: 10.1159/000515610 PMC8216018 34177417

[23] Gross R Zanoni M Seidel A Conzelmann C Gilg A Krnavek D. Heterologous ChAdOx1 nCoV-19 and BNT162b2 prime-boost vaccination elicits potent neutralizing antibody responses and T cell reactivity against prevalent SARS-CoV-2 variants. EBioMedicine (2021) 75:103761. doi: 10.1016/j.ebiom.2021.103761 34929493 PMC8682749

[24] Hoffmann M Arora P Gross R Seidel A Hornich BF Hahn AS. SARS-CoV-2 variants B.1.351 and P.1 escape from neutralizing antibodies. Cell (2021) 184(9):2384–93. doi: 10.1016/j.cell.2021.03.036 PMC7980144 33794143

[25] Hoffmann M Kruger N Schulz S Cossmann A Rocha C Kempf A. The omicron variant is highly resistant against antibody-mediated neutralization: implications for control of the COVID-19 pandemic. Cell (2022) 185(3):447–56. doi: 10.1016/j.cell.2021.12.032 PMC8702401 35026151

[26] Arora P Zhang L Rocha C Sidarovich A Kempf A Schulz S. Comparable neutralisation evasion of SARS-CoV-2 omicron subvariants BA.1, BA.2, and BA.3. Lancet Infect Dis (2022) 22(6):766–7. doi: 10.1016/S1473-3099(22)00224-9 PMC9005119 35427493

[27] Arora P Kempf A Nehlmeier I Schulz SR Cossmann A Stankov MV. Augmented neutralisation resistance of emerging omicron subvariants BA.2.12.1, BA.4, and BA.5. Lancet Infect Dis (2022) 22(8):1117–8. doi: 10.1016/S1473-3099(22)00422-4 PMC9239574 35777385

[28] Bruel T Hadjadj J Maes P Planas D Seve A Staropoli I. Serum neutralization of SARS-CoV-2 omicron sublineages BA.1 and BA.2 in patients receiving monoclonal antibodies. Nat Med (2022) 28(6):1297–302. doi: 10.1101/2022.03.09.22272066 35322239

[29] Yamasoba D Kosugi Y Kimura I Fujita S Uriu K Ito J. Neutralisation sensitivity of SARS-CoV-2 omicron subvariants to therapeutic monoclonal antibodies. Lancet Infect Dis (2022) 22(7):942–3. doi: 10.1016/S1473-3099(22)00365-6 PMC9179126 35690075

[30] Food and Drug Administration (FDA). Investigational COVID-19 convalescent plasma. Available at: https://www.federalregister.gov/documents/2023/03/13/2023-05094/guidance-documents-related-to-coronavirus-disease-2019-covid-19 (Accessed March 13, 2023).

[31] Korper S Gruner B Zickler D Wiesmann T Wuchter P Blasczyk R. One-year follow-up of the CAPSID randomized trial for high-dose convalescent plasma in severe COVID-19 patients. J Clin Invest (2022) 132(24):e163657. doi: 10.1172/JCI163657 36326824 PMC9753994

[32] Korley FK Durkalski-Mauldin V Yeatts SD Schulman K Davenport RD Dumont LJ. Early convalescent plasma for high-risk outpatients with covid-19. N Engl J Med (2021) 385(21):1951–60. doi: 10.1056/NEJMoa2103784 PMC8385553 34407339

[33] Rijnders BJA Huygens S Mitja O. Evidence-based dosing of convalescent plasma for COVID-19 in future trials. Clin Microbiol Infect (2022) 28(5):667–71. doi: 10.1016/j.cmi.2022.01.026 PMC8828382 35150881

[34] Bar KJ Shaw PA Choi GH Aqui N Fesnak A Yang JB. A randomized controlled study of convalescent plasma for individuals hospitalized with COVID-19 pneumonia. J Clin Invest (2021) 131(24):e155114. doi: 10.1172/JCI155114 34788233 PMC8670841

[35] RECOVERY Collaborative Group. Convalescent plasma in patients admitted to hospital with COVID-19 (RECOVERY): a randomised controlled, open-label, platform trial. Lancet (2021) 397(10289):2049–59. doi: 10.1101/2021.03.09.21252736 PMC8121538 34000257

[36] Avendano-Sola C Ramos-Martinez A Munez-Rubio E Ruiz-Antoran B Malo de MR Torres F. A multicenter randomized open-label clinical trial for convalescent plasma in patients hospitalized with COVID-19 pneumonia. J Clin Invest (2021) 131(20):e152740. doi: 10.1172/JCI152740 34473652 PMC8516461

[37] Estcourt LJ Turgeon AF McQuilten ZK McVerry BJ Al-Beidh F Annane D. Effect of convalescent plasma on organ support-free days in critically ill patients with COVID-19: a randomized clinical trial. JAMA (2021) 326(17):1690–702. doi: 10.1001/jama.2021.18178 PMC8491132 34606578

[38] Ortigoza MB Yoon H Goldfeld KS Troxel AB Daily JP Wu Y. Efficacy and safety of COVID-19 convalescent plasma in hospitalized patients: a randomized clinical trial. JAMA Intern Med (2022) 182(2):115–26. doi: 10.1001/jamainternmed.2021.6850 PMC8669605 34901997

[39] Biernat MM Kolasinska A Kwiatkowski J Urbaniak-Kujda D Biernat P Janocha-Litwin J. Early administration of convalescent plasma improves survival in patients with hematological malignancies and COVID-19. Viruses (2021) 13(3):436. doi: 10.3390/v13030436 33800528 PMC8001057

[40] Denkinger CM Janssen M Schakel U Gall J Leo A Stelmach P. Anti-SARS-CoV-2 antibody-containing plasma improves outcome in patients with hematologic or solid cancer and severe COVID-19: a randomized clinical trial. Nat Cancer (2023) 4(1):96–107. doi: 10.1038/s43018-022-00503-w 36581734 PMC9886549

[41] Lacombe K Hueso T Porcher R Mekinian An Chiarabini T Georgin-Lavialle S. Efficacy and safety of convalescent plasma to treat hospitalised COVID-19 patients with or without underlying immunodeficiency: a randomized clinical trial. medRxiv (2022). doi: 10.1101/2022.08.09.22278329

[42] Senefeld JW Franchini M Mengoli C Cruciani M Zani M Gorman EK. COVID-19 convalescent plasma for the treatment of immunocompromised patients: a systematic review and meta-analysis. JAMA Netw Open (2023) 6(1):e2250647. doi: 10.1001/jamanetworkopen.2022.50647 36633846 PMC9857047

[43] Salazar E Perez KK Ashraf M Chen J Castillo B Christensen PA. Treatment of coronavirus disease 2019 (COVID-19. patients with convalescent plasma. Am J Pathol (2020) 190(8):1680–90. doi: 10.1016/j.ajpath.2020.05.014 PMC7251400 32473109

[44] Marconato M Abela IA Hauser A Schwarzmuller M Katzensteiner R Braun DL. Antibodies from convalescent plasma promote SARS-CoV-2 clearance in individuals with and without endogenous antibody response. J Clin Invest (2022) 132(12):e158190. doi: 10.1172/JCI158190 35482408 PMC9197521

[45] Schmidt F Weisblum Y Muecksch F Hoffmann HH Michailidis E Lorenzi JCC. Measuring SARS-CoV-2 neutralizing antibody activity using pseudotyped and chimeric viruses. J Exp Med (2020) 217(11). doi: 10.1084/jem.20201181 PMC7372514 32692348

[46] Riepler L Rossler A Falch A Volland A Borena W von LD. Comparison of four SARS-CoV-2 neutralization assays. Vaccines (Basel (2020) 9(1):13. doi: 10.3390/vaccines9010013 33379160 PMC7824240

[47] Cox M Peacock TP Harvey WT Hughes J Wright DW Willett BJ. SARS-CoV-2 variant evasion of monoclonal antibodies based on *in vitro* studies. Nat Rev Microbiol (2023) 21(2):112–24. doi: 10.1038/s41579-022-00809-7 PMC9616429 36307535

[48] Wang Z Muecksch F Schaefer-Babajew D Finkin S Viant C Gaebler C. Naturally enhanced neutralizing breadth against SARS-CoV-2 one year after infection. Nature (2021) 595(7867):426–31. doi: 10.1038/s41586-021-03696-9 PMC8277577 34126625

